# 

**Published:** 1838-01-31

**Authors:** 


### BOOKS RECEIVED.

1. The Philadelphia Practice of Midwifery. By C. D. Meigs, M. D. Philadelphia, 1838.
2. An Oration on the Improvements on Medicine. By Joseph Warrington, M. D.
3. An Introductory Lecture, delivered at the opening of the session of the Medical College of the State of S. Carolina, on the second Monday of November, 1837. By E. Geddings, M. D., Professor of Pathology, and Medical Jurisprudence, &c. Published by the Class, 8vo pp. 38. Charleston, 1838.
4. A Clinical Lecture on the Primary Treatment of Injuries, delivered at the New York Hospital, by A. H. Stevens, M. D. Nov. 22d, 1837.
5. The American Journal of the Medical Sciences, Nov. 1837. (In exchange.)
6. The Select Medical Library and Eclectic Journal of Medicine. Edited by J. Bell, M. D., &c. &c. Published Monthly, vol. 2, Nos. 1, 2 and 3, for Nov. Dec. and Jan. 1837-8. (In exchange.)
7. The Boston Medical and Surgical Journal Nos. 23, 24 and 25 for Jan. 1838. (In exchange.)

### TERMS.

The Examiner is published every alternate Wednesday, at *Three dollars* per annum, *payable in every instance in advance*. Two copies, if sent to the same address, for Five dollars.

Persons disposed to subscribe, will please send their address by mail, *accompanied with a remittance*.

Letters and communications may be addressed to the "Editors of the Medical Examiner," and must be *post paid, otherwise they will not be taken from the office*.

No subscriptions received for less than one year, commencing January 3d, 1838.

